# Euterpe music therapy method for children with cerebral palsy

**DOI:** 10.3389/fneur.2024.1388712

**Published:** 2024-04-10

**Authors:** Tommaso Liuzzi, Sarah Bompard, Massimiliano Raponi, Fiammetta D’Arienzo, Susanna Staccioli, Eleonora Napoli, Martina Frascari Diotallevi, Simone Piga, Roberto Giuliani, Enrico Castelli

**Affiliations:** ^1^Unit of Neurorehabilitation, Bambino Gesù Children’s Hospital, IRCCS, Rome, Italy; ^2^Santa Cecilia Conservatory of Music, Rome, Italy; ^3^Health Directorate, Bambino Gesù Children’s Hospital, IRCCS, Rome, Italy; ^4^Euterpe APS Cultural Association, Rome, Italy; ^5^Unit of Epidemiology, Bambino Gesù Children’s Hospital, IRCCS, Rome, Italy

**Keywords:** music therapy, cerebral palsy, neurorehabilitation, Euterpe method, sleep disturbances, quality of life, parental distress, children

## Abstract

**Introduction:**

The main purpose of our study was to evaluate whether involvement in a personalized music therapy program (Euterpe method), could improve the condition of children with cerebral palsy and their parents, compared to a control group. It investigated whether it could positively affect children’s sleep quality, temperament and quality of life, quality of family life, and parental stress.

**Methods:**

A prospective single-center experimental study was conducted at “Bambino Gesù” Children’s Hospital (Rome, Italy). All subjects involved attended an intensive rehabilitation program in the Neurorehabilitation Unit. In a group of patients (*n* = 25), a music therapy treatment was applied to evaluate the effect before and after the intervention. This group was also compared with a control group (*n* = 10) undergoing a standard protocol without music therapy.

**Results:**

In the experimental group, the analysis shows statistically significant effects in the Disorders of initiating and maintaining sleep (*p* = 0.050) and the Sleep wake transition disorders (*p* = 0.026) factors, and the total score (*p* = 0.031) of Sleep Disturbances Scale for Children; the Positive emotionality scale (*p* = 0.013) of Italian Questionnaires of Temperament (QUIT); the Emotional Functioning (*p* = 0.029), Social Functioning (*p* = 0.012), Worry (*p* = 0.032), Daily Activities (*p* = 0.032), Total Score (*p* = 0.039) and Parent HRQL Summary Score (*p* = 0.035) dimensions of Pediatric Quality of Life for family. While in the control group, only the Attention scale of QUIT (*p* = 0.003) reaches statistical significance.

**Discussion:**

Our study suggests that music therapy with the Euterpe Method has beneficial effects on fundamental aspects of the child’s and his parents’ lives, such as sleep, emotion control, and quality of family life.

## 1 Introduction

Cerebral palsy (CP) is a neurodevelopmental condition that begins in early childhood and persists throughout life. It is defined as a group of permanent disorders of the development of movement and posture, causing activity restriction that is attributed to non-progressive lesions occurring in the developing fetal or infant brain. Motor disorders in CP are often accompanied by sensory, perceptual, cognitive, communication, and behavioral disorders, epilepsy, and secondary musculoskeletal difficulties (1). In this context, multisensory stimulation interventions may improve sensory and motor function (2). A systematic literature review found that visual-perceptual impairment ranged from 40% to 50% in children with CP (3), as also confirmed by Rauchenzauner et al. (4), who found visual-perceptual impairment in 59.5% of them. Novak also reports that 4% of children with CP have severe hearing problems or are deaf (5). Motor learning is frequently impaired by deficits in sensory information processing (6). Both sensory and perceptual disturbances can be directly related to the CP etiology or the result of a child’s limited activities, experiences, and learning (7).

The recent review by de Almeida et al. found that muscle spasms, contractures, and reduced ability to change body position at night in children with CP interfere with sleep quality. The studies reviewed highlighted the severity of sleep disorders and report an incidence of the population with CP ranging from 23.4% to 46% (8).

Interventions for children with CP include rehabilitation treatments, medical therapies, and orthopedic surgery (9). Castelli et al. explained that rehabilitation is a complex process aimed at promoting the best possible participation and quality of life for the child and family (10). Moreover, early auditory-tactile-visual-vestibular rehabilitation improved motor and cognitive performance in children with severe brain injury or extreme prematurity, as described by Nelson et al. (11). Holt and Mikati comprehensively reviewed the effects of early rehabilitation to improve the functional development of infants with perinatal brain damage. They concluded that exposure to enriched sensory environments can improve cognitive outcomes and increase brain growth (12).

In addition to conventional rehabilitation, music therapy can help achieve the treatment goals.

The World Federation of Music Therapy, after the absorption of several associations, based on international experiences has defined music therapy as *“the professional use of music and its elements as an intervention in medical, educational, and everyday environments with individuals, groups, families, or communities who seek to optimize their quality of life and improve their physical, social, communicative, emotional, intellectual, and spiritual health and wellbeing. Research, practice, education, and clinical training in music therapy are based on professional standards according to cultural, social, and political contexts”* (13).

Since 1944, when the first-degree course was held at Michigan State College in the United States, music therapy has had a constant and ever-increasing development toward scientific research and clinical therapy. However, it was in the 1990s that the live observation provided by neuroimaging techniques led to the birth of neurological music therapy. Diagnostic imaging techniques have provided research, that studies and correlates brain activity and music, with demonstrations of significant effects on the stimulation of physiologically complex cognitive, affective, and sensorimotor processes (14). These findings are an important therapeutic aid for the treatment of children with CP.

The study of Alves-Pinto et al. investigated music-supported therapies for the rehabilitation of motor disorders. Their findings showed that actively playing a musical instrument can be an effective means of promoting the coordination of hand movements and, more generally, triggering the neuroplastic processes necessary for the development of sensorimotor skills in patients with early brain damage (15). Ghai et al. (16) analyze the effects of rhythmic auditory cueing on gait in people with CP. Their review suggests evidence for applying rhythmic auditory cueing to enhance gait performance and stability. Motor function in CP people has also been studied by Kantor et al. (17) in correlation with the effects of vibroacoustic therapy. They stated a statistically significant improvement in children’s and adult’s range of motion. Marrades-Caballero et al. (18), investigating the impact of an optimized music therapy program including active music techniques on the function of children with CP, observed significant improvements in the overall and specific “arm and hand position” as well as “activities.” All of these improvements persisted after 4 months. The study by Coppola et al. highlights the reduction of epileptic seizures following the administration of Mozart’s sonata for two pianos in D major, K448. The authors did not study the implications of sleep, but parents reported observations of improved sleep and daytime behavior (19).

Since the effect of music therapy has been studied in CP children mainly about motor aspects or training with musical instruments, our study wanted to investigate different critical aspects of the child’s life. De Almeida et al. report the need to conduct scientific research to investigate the problem of sleep quality related to quality of life in children with CP and their families (8).

Our study is the first aimed to evaluate whether a personalized music therapy program according to the Euterpe Method (EM), conducted by a certified music therapist, can positively affect the sleep quality, temperament, and quality of life of children with CP, the quality of family life, and parental stress.

## 2 Materials and methods

### 2.1 Study design

A prospective, single-center, experimental, study was conducted at the “Bambino Gesù” Children’s Hospital (Rome, Italy) between May 2021 and August 2022.

All subjects involved in the study attended an intensive rehabilitation program in the Neurorehabilitation Unit of the Hospital as in-patients. According to the child’s needs, the rehabilitation takes place daily, 3 h a day, 6 days a week. It includes physiotherapy, neuromotor rehabilitation, speech therapy, occupational therapy, psychomotor therapy, neuro-visual and communicative rehabilitation.

Enrollment criteria were as follows: age between 0 and 10 years, diagnosis of CP according to Morgan et al. (20), and in-hospital stay between 3 and 4 weeks for intensive rehabilitation. Children with severe hearing problems or deafness were excluded.

We included children with CP younger than 10 years of age because afterward, patients have a higher possibility of presenting with secondary impairments and complications that worsen function or interfere with learning. Pain secondary to musculoskeletal problems is less frequent in children than in adolescents (21). Furthermore, in older children with CP, excessive knee flexion and crouch gait are common (22). Throughout adulthood, a gradual decline in functional ability has been reported across all Gross Motor Functional Classification System levels (GMFCS) (23). Moreover, early life is the period with the highest potential to counteract negative sequelae, given the high plasticity of the young brain (24). We included children with CP because it is the leading cause of chronic disability in pediatric patients (25). Considering that in our department children undergo music therapy three times a week and, according to our experience, it takes about eight sessions to achieve the therapeutic goals. For these reasons, we have included only patients who have been hospitalized for at least 3 weeks. We used the GMFCS to assess their functional status (26). The GMFCS is a 5-level functional classification that differentiates children and youth with CP according to their current gross motor abilities. Children with GMFCS level I can generally walk without restrictions, while those classified as level V are usually very limited in their ability to move themselves.

Informed consent was provided to the parents of the children who met the inclusion criteria of the study. All parents received and autonomously completed the parent-report questionnaires for assessment at T0 at the time of inclusion in the study.

Following randomization, An EM music therapy treatment was administered to a group of children (experimental group—EG), to assess the effect before and after intervention. The same group was compared with a control group (CG). This latter underwent a rehabilitation program without music therapy.

All children included in the study were hospitalized for intensive rehabilitation and returned home only upon discharge. Therefore, there could be no interference with other music therapy programs carried out at home or with listening to ambient music.

EG and CG parents, whose children had completed the rehabilitation pathway, autonomously filled out the parent-report questionnaires at T1, 3 weeks after T0.

The outcome assessor was a psychologist who was blinded to the assignment of children to one of the two groups (randomization) and the scoring of questionnaires completed by parents. The psychologist was always the same person and did not intervene in the compilation process.

**Figure 1:**
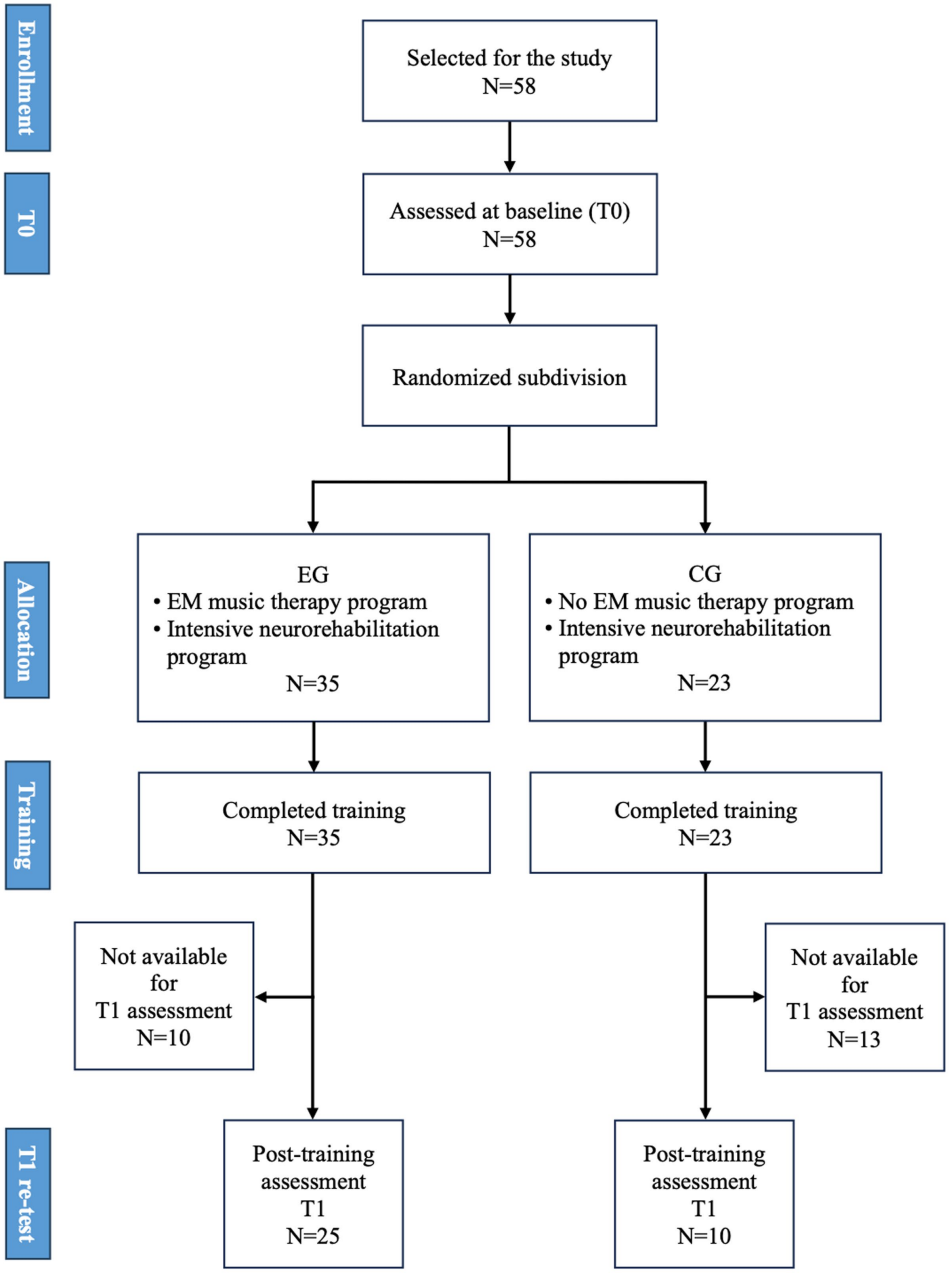
Study design. Enrollment procedure and flow of study procedure.

The whole process is summarized in the flowchart (Figure 1).

### 2.2 Euterpe method’s music therapy

The EM is a type of music therapy in which music is associated with sensory stimulation, following specific algorithms and procedures.

Therapy is aimed at the child and requires the involvement and active participation of the parent. The activity is called Sound in Multisensory Stimulation. These are complex sensory and cross-modal stimulations using elaborate sounds and personalized compositions in osmosis with the environment.

In this study, *EM active* music therapy was used, which is the most operative modality of the methodology. It is carried out in a logical consequential succession of *“sound actions”* and therapeutic activities aimed at achieving the clinical goals established by the medical team.

The EM music therapy protocol was designed based on the clinical needs of the intensive neurorehabilitation program. This program is carried out in a minimum hospitalization of 3 weeks, to have at least 8 EM sessions with the child and parent, plus the “sound anamnesis” session.

EM active starts with the sound anamnesis called *“personal sound history,”* and the creation of a personalized database consisting of four archives.

The personal sound history is divided into two parts: the first is related to the collection of clinical, sensory, and musical information; the second consists of the recording of the mother’s voice.

The archives contain: (A) clinical data, categorizations of the different sounds; (B) videos of the therapy sessions; (C) significant photos of the therapeutic pathway; (D) cards describing the activities carried out in the different treatment areas.

The EM training session follows a specific succession of steps, which begin with the “welcoming phase” of the patient and parent within the multisensory space.

The “*compositional sound interventions”* are a process of steps that begin with the reduction of background noise on the raw track of the mother’s voice. As a result, the track is fragmented into audio clips that are named and stored according to categorizations (e.g., prosodies of the child’s name, endearments, positive reinforcements, lullabies, songs, etc.). The mother’s pre-recorded sound allows us, from the first therapy, to send the audio clips to the different sources of propagation (loudspeakers). In addition, of considerable importance, the pre-recorded sound can be simultaneously superimposed on the speech of the mother addressing her baby. This intervention is mainly conducted in order to motivate and direct the patient toward the sound source and promote mother–child emotional interaction.

**Figure 2:**
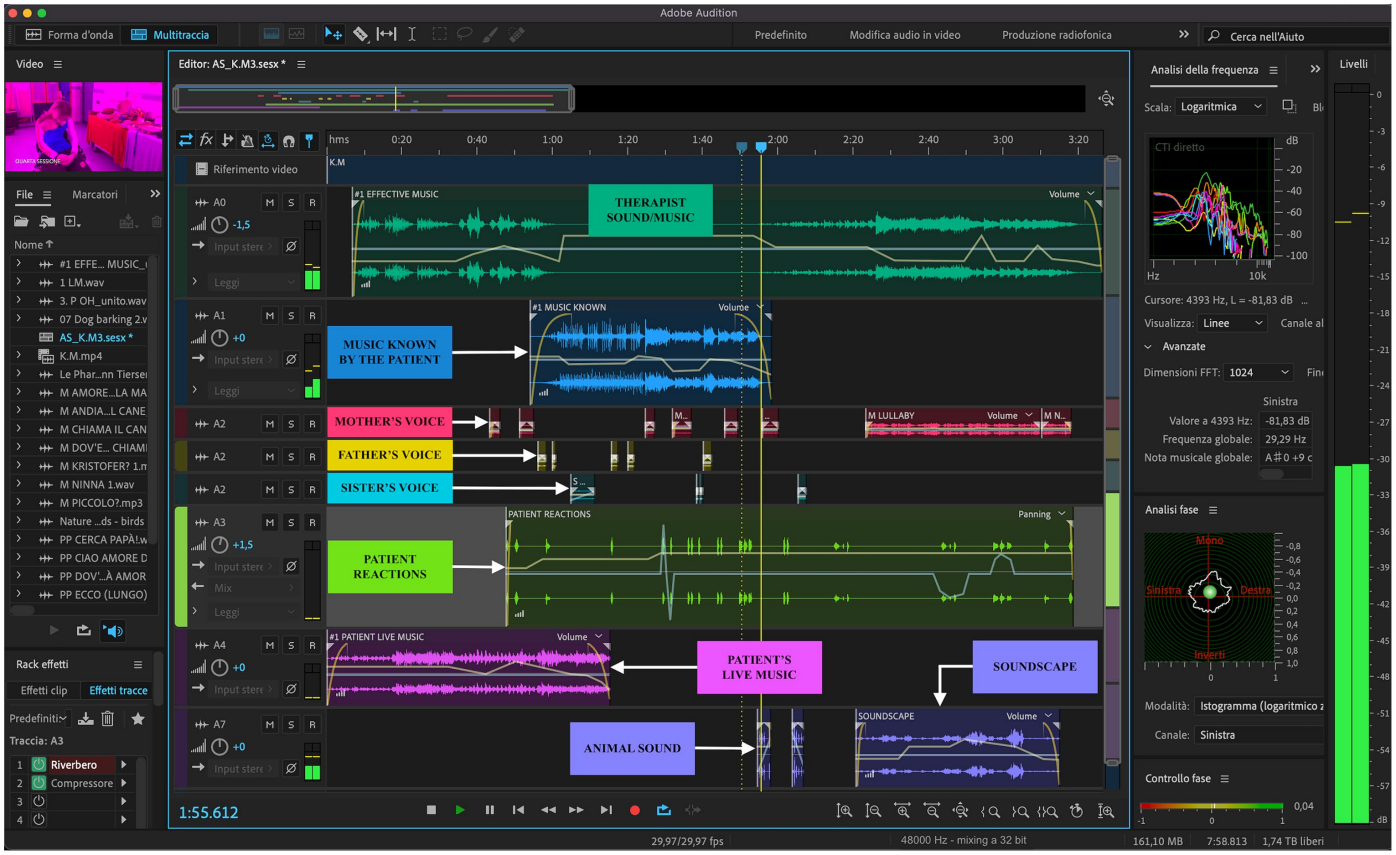
An example of a custom composition. The left axis of the image shows the edited video clip of the sessions, the library of audio clips, the effects applied to tracks, and at the bottom are indicated the start, end, and duration of the project; at the top, there is the timeline expressed in decimal time format (mm:ss.ddd); on the right axis there are the Frequency Analysis, the Phase Analysis, the Phase Control, and the Level Meter; the bottom shows the Mixer and the effect panels, and finally, the Sample Type in the status bar indicates the 48,000 Hz sample rate, the 32-bit depth, and the buffer size of 1,024.

The following are the steps in a succession of the “compositional sound interventions” and the aims: (1) the instrumental or vocal live has as its main purpose the observation of the child’s reactions; (2) the recorded stimuli are re-proposed, and elaborated adapting them to the psychophysiological condition of the patient, in order to activate and motivate the child to interact with the therapeutic action; (3) the administration of effective clips (e.g., edited child and mother’s voices, known music, soundscape, and landscape), is intended to activate visual, motor, and emotional areas; (4) finally, the personalized composition is made with the overlapping of all the clips that are most effective for the therapeutic objectives (see Figure 2).

In support of the effects of this musical procedure, we cite the work of Koelsch. He states that the therapeutic action of music involves brain structures in cognitive, sensorimotor, and emotional processing. In addition, he asserts that music involves sensory processes, attention, memory-related processes, and perception-action mediation (“mirror neuron system” activity). Finally, he hypothesizes that the activation of these processes by music may have beneficial effects on the psychological and physiological health of individuals, although the mechanisms underlying these effects are currently not well understood (27). Similarly, Magee et al. (28) also state that interventions with music are used in rehabilitation to stimulate brain functions involved in movement, cognition, language, emotions, and sensory perceptions.

A fundamental prerogative is that EM music therapy takes place in a personalized and soundproofed multisensory space, named “synesthesia room.” It includes multiple devices: LED projectors, moving heads and strips; a sound diffusion system with mixer and audio interface; several laptops; camcorders; vibrating devices; aroma diffusers; postural instruments; tactile, visual, and olfactory paths; sensors, touch monitors; instruments, and musical objects; and interactions with image projections through augmented reality. In support of EM music therapy, which takes place in a designed space (synesthesia room), we mention Serino’s study. In fact, he affirms that *“The brain represents the space to perceive and interact with external stimuli in the environment”* (29). Neuroimaging has shown that multiple representations of space built by the brain are related to specific reference frames. The latter depend on the source of sensory stimulation and nature of the interaction between the individual and environment (30). In this way, Andersen et al. show how multimodal sensory signals, including vision, somatosensation, audition, and vestibular sensation, converge in the posterior parietal cortex to encode the spatial positions of targets for movement. These signals are combined using a specific mechanism that composes coordinate frames of the various input signals into spatial representations. Within these spatial representations of the posterior parietal cortex are neural activities related to higher cognitive functions, including attention. Thus, there is an intermediate stage between sensory and motor structures that contains an abstract representation of space and the mental operations important for planning movements (31).

Thus, stimulation can be harmonized in a resilient way to the different needs of children and to specific therapeutic objectives previously established by the medical team.

The EM music therapy sessions take place three times a week in the afternoon following the intensive rehabilitation therapies, last 35/40 min each, and are held by a certified professional. In our case, he has two postgraduate Master’s degrees in Music Therapy and Autism Spectrum Disorder and three Master’s degrees in Orchestral Conducting, Clarinet, and Management in Educational Services.

The objectives of these therapies are: to improve the child’s quality of sleep, temperament, and ability to interact with the environment, the relationship between parents and children, and to support mothers’ psychological stress. In addition, they are aimed at enhancing the quality of life of both.

### 2.3 Participants

From a large cohort, we selected 58 children according to the inclusion criteria. They were divided into 2 groups according to randomization, 35 in the EG and 23 in the CG.

Thirty-five children with CP (22 males and 13 females) were selected and underwent music therapy. Twenty-five children (15 males and 10 females) completed the assessment both at T0 and T1 and were included in the EG.

Twenty-three children with CP (11 males and 12 females) of the same age and clinical features of EG were enrolled. They did not attend music sessions but rehabilitative treatments analogous to the children of the EG. Ten children with CP (4 males and 6 females) completed T0 and T1 assessments and were included in the CG.

This study was performed following the Declaration of Helsinki, and informed consent was provided to parents.

For an overview of group characteristics and test statistics, see Figure 1 and Table 1.

**Table 1:** Descriptive characteristics of the two groups.

		EG (*n* = 25)	CG (*n* = 10)	*P*
Children’s variables	Age in groups (years)			0.081^a^
	0–5	21 (84.0)	5 (50.0)	
	>5	4 (16.0)	5 (50.0)	
	Gender, *N* (%),			
	Male	15 (40.0)	4 (40.0)	0.454^a^
	Female	10 (60.0)	6 (60.0)	
	GMFCS, m ± SD	4.0 ± 1.0	3.4 ± 1.4	0.190^b^
	EM Sessions^*^, m ± SD	7.5 ± 2.5	-	-

^a^Result of a Fisher exact test. ^b^Result of a two-sample *t*-test. ^*^The cell is empty because the CG has not carried out the EM music therapy program.

### 2.4 Measures

All children’s parents involved in the study (EG and CG) autonomously completed five parent-report questionnaires:

- The Sleep Disturbance Scale for Children (SDSC) (32) is a parent questionnaire used to investigate the quality of sleep among children. In our analysis we use total score and scores obtained in each subscale (DIMS, disorders of initiating and maintaining sleep; SBD, sleep breathing disorders; DA, disorders of arousal/nightmares; SWTD, sleep–wake transition disorders; DOES, disorders of excessive somnolence; SHY, sleep hyperhidrosis).
- The use of Italian Questionnaires of Temperament (QUIT) (33) highlights the child’s behavior in interaction with others, when playing and concerning news. QUIT analyzes several domains: social orientation, inhibition to novelty, motor activity, positive emotionality, negative emotionality, and attention. According to the test authors, individual behavioral reactivity to environmental stimuli (as. emotional, attentional, and motor activity) and self-regulation in childhood represent the fundamental components of temperament. The way temperament is expressed is influenced by the environment and life experience (34).
- To investigate the health-related quality of life of children and mothers, we administered the Pediatric Quality of Life. According to age ranges, we dispensed the parent-report questionnaire for infants (PEDSQL-C) (35), toddlers, and children (PEDSQL-C) (36), and finally the family impact module (PEDSQL-F) (37). In our analysis, we considered all dimensions scores, total scores, and summary scores obtained in different modules. The Parent Report for Infants (used for children ages 1–24 months) comprises 5 dimensions: Physical Functioning (PF), Physical Symptoms (PS), Emotional Functioning (EF), Social Functioning (SF), and Cognitive Functioning (CF). The Parent Report for Toddlers (used for children ages 2–4) and the Parent Report for Children comprise 4 dimensions: Physical Functioning (PF), Emotional Functioning (EF), Social Functioning (SF), and Cognitive Functioning (CF). Parent Report modules for Infants, Toddlers, and Children provide, in addition to scores in individual domains, the Total Score (TOT − sum of all items) and two Summary Scores: Psychosocial Health (PsyHSS) and Physical Health (PhyHSS). The Family Impact Module comprises 8 dimensions: Physical Functioning (PF), Emotional Functioning (EF), Social Functioning (SF), Cognitive Functioning (CF), Communication (Com), Worry (W), Daily Activities (DA), and Family Relationships (FR). In addition to scores in individual domains, this module also provides the Total Score (TOT − sum of all items) and two Summary Scores: Parent HRQL and Family Functioning (FFSS).
- The Parenting Stress Index-Short Form (PSI-SF) (38) was used to investigate parenting stress. In our analysis, we include the total score and its sub-scores (PD, parental distress; PDCI, parent–child dysfunctional interaction; DC, difficult child; DR, defensive responding).

### 2.5 Statistical analysis

All statistical analyses were performed using STATA, Statistical Software: Release 17 (StataCorp LP, College Station, TX). The Shapiro–Wilk test was used to assess the normality of the data. Categorical variables were summarized by absolute frequencies and percentages, and continuous variables by mean and standard deviation SD.

To determine statistical differences for all scores between baseline (T0) and post-music therapy (T1) the T-test for matched data was used for continuous variables. Furthermore, scores between subjects in the EG and CG were compared at baseline and after music therapy with T-test for independent samples, and categorical variables were compared with Chi-square or Fisher exact test. The statistical significance was set at *p* < 0.05.

## 3 Results

### 3.1 Sample’s descriptive characteristics

The baseline comparison of both groups shows the same structural characteristics, i.e., without statistically significant differences.

Gender was analyzed with the Fisher exact test. The sample shows no statistically significant differences (*p* = 0.454).

Even in terms of the assessment of functional status carried out by GMFCS, the difference analyzed with a two-sample t-test is not statistically significant (*p* = 0.190).

Age analysis was performed using the Fisher exact test. Also concerning age classifications, the two groups do not differ at baseline (*p* = 0.081).

Thus, EG and CG were homogeneous in the distribution by gender, age, and GMFCS.

### 3.2 Results of the experimental group

There are statistically significant improvements compared to pre- and post-EM music therapy treatment (Table 2).

**Table 2:** Scores of the experimental group (results are reported as mean ± standard deviation).

		T0	T1	*P*
Sleep Disturbances Scale for Children (SDSC)	Disorders of initiating and maintaining sleep (DIMS)	**14.6 ± 7.1**	**13.4 ± 6.1**	**0.050**
	Sleep breathing disorders (SBD)	4.4 ± 2.4	4.4 ± 2.4	1.000
	Disorders of arousal (DA)	3.1 ± 1.6	2.9 ± 1.0	0.519
	Sleep wake transition disorders (SWTD)	**9.6 ± 3.3**	**8.0 ± 2.9**	**0.026**
	Disorders of excessive somnolence (DOES)	7.1 ± 5.6	5.6 ± 1.9	0.195
	Sleep hyperhidrosis (SHY)	3.5 ± 2.4	3.4 ± 1.8	0.722
	Sleep Disturbance Scale for Children (SDSC) total score	**42.4 ± 14.5**	**37.7 ± 12.7**	**0.031**
Italian Questionnaires of Temperament (QUIT)	Social orientation	3.8 ± 1.3	3.7 ± 1.2	0.693
	Inhibition to novelty	3.4 ± 0.9	3.1 ± 1.2	0.195
	Motor activity	3.3 ± 0.9	3.3 ± 0.8	0.950
	Positive emotionality	**4.1 ± 1.4**	**4.4 ± 1.2**	**0.013**
	Negative emotionality	2.7 ± 1.0	2.4 ± 0.8	0.076
	Attention	3.8 ± 0.9	3.8 ± 0.9	0.932
Pediatric Quality of Life 2.0 Family Impact Module (PEDSQL-F)	Physical Functioning (PF)	58.0 ± 20.2	61.2 ± 20.8	0.179
	Emotional Functioning (EF)	**61.6 ± 26.4**	**69.6 ± 22.6**	**0.029**
	Social Functioning (SF)	**61.5 ± 25.4**	**68.3 ± 26.4**	**0.012**
	Cognitive Functioning (CF)	72.8 ± 21.3	73.6 ± 18.2	0.730
	Communication (Com)	72.9 ± 20.1	73.0 ± 21.6	0.966
	Worry (W)	**45.2 ± 24.6**	**52.4 ± 21.5**	**0.032**
	Daily Activities (DA)	**39.3 ± 26.3**	**46.0 ± 24.7**	**0.032**
	Family Relationships (FR)	78.2 ± 22.5	77.8 ± 23.5	0.877
	Total Score (TOT)	**61.5 ± 18.6**	**65.5 ± 17.9**	**0.039**
	Parent HRQL Summary Score (HRQL)	**63.1 ± 19.6**	**67.5 ± 19.2**	**0.035**
	Family Functioning Summary Score (FFSS)	63.6 ± 21.0	65.9 ± 22.2	0.247
Pediatric Quality of Life (PEDSQL-C)	Physical Functioning (PF)	41.6 ± 22.8	46.7 ± 24.7	0.297
	Physical Symptoms (PS)	59.2 ± 15.9	62.8 ± 16.8	0.325
	Emotional Functioning (EF)	67.0 ± 18.1	71.7 ± 23.9	0.155
	Social Functioning (SF)	55.1 ± 27.3	60.5 ± 27.3	0.291
	Cognitive Functioning (CF)	46.5 ± 37.8	32.5 ± 37.2	0.283
	School Functioning (SchF)	52.8 ± 21.5	56.9 ± 18.6	0.456
	Psychosocial Health Summary Scale (PsyHSS)	59.6 ± 19.0	62.5 ± 20.8	0.329
	Physical Health Summary Scale (PhyHSS)	42.9 ± 21.8	48.6 ± 25.0	0.187
	Total Score (TOT)	52.6 ± 16.7	56.7 ± 18.5	0.135
Parenting Stress Index-Short Form (PSI-SF)	Parental Distress (PD)	56.2 ± 28.2	55.4 ± 31.5	0.857
	Parent–Child Dysfunctional Interaction (PCDI)	63.4 ± 22.0	60.2 ± 23.6	0.466
	Difficult Child (DC)	53.8 ± 34.7	54.0 ± 32.9	0.961
	Defensive Responding (DR)	49.2 ± 30.2	47.4 ± 30.1	0.633
	Parenting Stress Index-Short Form (PSI-SF) total score	58.6 ± 30.2	57.6 ± 29.4	0.833

Statistically significant values are formatted in bold.

The analysis of the SDSC shows statistically significant effects in the DIMS factor (*p* = 0.050), the SWTD factor (*p* = 0.026), and the SDSC total score (*p* = 0.031). Other factors (DA, DOES, and SHY) improved but not significantly, while the SBD factor mean remained unchanged M ± SD (T0) 4.4 ± 2.4; M ± SD (T1) 4.4 ± 2.4.

Referring to QUIT, the positive emotionality scale (*p* = 0.013) shows a statistically significant score, while the other scales (inhibition to novelty and negative emotionality) show improvements that, however, are not significant. The social orientation, motor activity, and attention scales also present a result that does not improve.

The PEDSQL-F has a general improvement (PF, CF, Comm, and FFSS), specifically in the EF (*p* = 0.029), SF (*p* = 0.012), W (*p* = 0.032), DA (*p* = 0.032), TOT (*p* = 0.039) and HRQL (*p* = 0.035) dimensions. On the other hand, the value of FR does not improve statistically.

### 3.3 Results of the control group

The statistical analysis between T0 and T1 of CG is shown in Table 3.

**Table 3:** Scores of the control group (results are reported as mean ± standard deviation).

		T0	T1	*P*
Sleep Disturbances Scale for Children (SDSC)	Disorders of initiating and maintaining sleep (DIMS)	12,7 ± 4,1	12.3 ± 3.1	0.626
	Sleep breathing disorders (SBD)	6.1 ± 7.4	3.9 ± 1.0	0.346
	Disorders of arousal (DA)	4.0 ± 2.2	3.7 ± 1.3	0.468
	Sleep wake transition disorders (SWTD)	10.4 ± 4.7	9.4 ± 2.3	0.497
	Disorders of excessive somnolence (DOES)	5.2 ± 1.4	5.0 ± 1.3	0.443
	Sleep hyperhidrosis (SHY)	2.5 ± 1.1	2.4 ± 1.0	0.847
	Sleep Disturbance Scale for Children (SDSC) total score	40.9 ± 11.3	36.7 ± 7.4	0.095
Italian Questionnaires of Temperament (QUIT)	Social orientation	4.4 ± 1.1	4.2 ± 1.1	0.643
	Inhibition to novelty	2.3 ± 0.4	2.5 ± 0.8	0.238
	Motor activity	3.0 ± 1.1	2.9 ± 1.0	0.493
	Positive emotionality	5.2 ± 0.6	4.9 ± 0.8	0.325
	Negative emotionality	2.5 ± 0.6	2.2 ± 0.7	0.200
	Attention	**4.0 ± 1.0**	**4.4 ± 1.0**	**0.003**
Pediatric Quality of Life 2.0 Family Impact Module (PEDSQL-F)	Physical Functioning (PF)	70.4 ± 14.1	80.4 ± 11.8	0.057
	Emotional Functioning (EF)	71.5 ± 21.4	80.0 ± 20.3	0.090
	Social Functioning (SF)	73.8 ± 25.5	80.0 ± 18.6	0.117
	Cognitive Functioning (CF)	82.5 ± 18.4	78.0 ± 24.3	0.171
	Communication (Com)	76.7 ± 25.7	85.8 ± 18.4	0.066
	Worry (W)	52.5 ± 25.7	57.5 ± 28.2	0.252
	Daily Activities (DA)	61.7 ± 24.3	60.0 ± 21.8	0.509
	Family Relationships (FR)	82.0 ± 17.2	82.0 ± 17.4	1.000
	Total Score (TOT)	71.5 ± 17.7	75.8 ± 15.6	0.138
	Parent HRQL Summary Score (HRQL)	74.4 ± 17.1	79.6 ± 14.8	0.119
	Family Functioning Summary Score (FFSS)	74.4 ± 17.1	73.8 ± 15.0	0.825
Pediatric Quality of Life (PEDSQL-C)	Physical Functioning (PF)	44.4 ± 28.1	46.6 ± 30.6	0.382
	Physical Symptoms (PS)^*^	-	-	-
	Emotional Functioning (EF)	78.0 ± 8.6	76.0 ± 13.3	0.606
	Social Functioning (SF)	63.5 ± 16.0	68.0 ± 19.9	0.378
	Cognitive Functioning (CF)^*^	-	-	-
	School Functioning (SchF)	67.7 ± 20.3	72.8 ± 22.1	0.139
	Psychosocial Health Summary Scale (PsyHSS)	70.3 ± 11.1	72.4 ± 16.1	0.526
	Physical Health Summary Scale (PhyHSS)	44.4 ± 28.1	46.6 ± 30.6	0.382
	Total Score (TOT)	61.1 ± 15.4	63.2 ± 19.8	0.449
Parenting Stress Index-Short Form (PSI-SF)	Parental Distress (PD)	46.6 ± 36.8	42.6 ± 35.7	0.525
	Parent–Child Dysfunctional Interaction (PCDI)	32.1 ± 25.4	41.1 ± 22.6	0.134
	Difficult Child (DC)	48.7 ± 29.5	41.6 ± 29.8	0.433
	Defensive Responding (DR)	45.1 ± 37.4	39.1 ± 35.6	0.292
	Parenting Stress Index-Short Form (PSI-SF) total score	42.1 ± 29.1	41.1 ± 31.9	0.832

Statistically significant values are formatted in bold. ^*^The empty PS and CF cells of the PEDSQL-C correspond to dimensions that cannot be evaluated in the age range of the CG study population.

We observe an improvement in the trend in several variables but it does not reach statistical significance except for the attention scale of the QUIT (*p* = 0.003).

The following scales do not show statistically significant effects: PCDI of the PSI; social orientation, inhibition to novelty, and positive emotionality of the QUIT; CF, DA, and FFSS of the PEDSQL-F; EF of the PEDSQL-C.

Moreover, the trend is unchanged in the FR dimension of the PEDSQL-F, the mean remains the same value while the standard deviation has an irrelevant change M ± SD (T0) 82.0 ± 17.2; M ± SD (T1) 82.0 ± 17.4.

### 3.4 Differences between the two groups

The comparison of the results between the two groups at T0 did not highlight statistically significant differences. The comparison of both groups at T1 detects relevant changes. The analysis of the comparison between the EG and the CG at T1 shows statistically significant changes in the following scales: the PCDI scale (*p* = 0.036) of PSI, and the PF (*p* = 0.010) dimension of the PEDSQL-F (Table 4).

**Table 4:** Post-treatment comparison EG VS CG (results are reported as mean ± standard deviation).

		EG (T1)	CG (T1)	*P*
Sleep Disturbances Scale for Children (SDSC)	Disorders of initiating and maintaining sleep (DIMS)	13.4 ± 6.1	12.3 ± 3.1	0.583
	Sleep breathing disorders (SBD)	4.4 ± 2.4	3.9 ± 1.0	0.536
	Disorders of arousal (DA)	2.9 ± 1.0	3.7 ± 1.3	0.060
	Sleep wake transition disorders (SWTD)	8.0 ± 2.9	9.4 ± 2.3	0.184
	Disorders of excessive somnolence (DOES)	5.6 ± 1.9	5.0 ± 1.3	0.410
	Sleep hyperhidrosis (SHY)	3.4 ± 1.8	2.4 ± 1.0	0.100
	Sleep Disturbance Scale for Children (SDSC) total score	37.7 ± 12.7	36.7 ± 7.4	0.814
Italian Questionnaires of Temperament (QUIT)	Social orientation	3.7 ± 1.2	4.2 ± 1.1	0.242
	Inhibition to novelty	3.1 ± 1.2	2.5 ± 0.8	0.179
	Motor activity	3.3 ± 0.8	2.9 ± 1.0	0.196
	Positive emotionality	4.4 ± 1.2	4.9 ± 0.8	0.231
	Negative emotionality	2.4 ± 0.8	2.2 ± 0.7	0.389
	Attention	3.8 ± 0.9	4.4 ± 1.0	0.107
Pediatric Quality of Life 2.0 Family Impact Module (PEDSQL-F)	Physical Functioning (PF)	**61.2 ± 20.8**	**80.4 ± 11.8**	**0.010**
	Emotional Functioning (EF)	69.6 ± 22.6	80.0 ± 20.3	0.216
	Social Functioning (SF)	68.3 ± 26.4	80.0 ± 18.6	0.209
	Cognitive Functioning (CF)	73.6 ± 18.2	78.0 ± 24.3	0.557
	Communication (Com)	73.0 ± 21.6	85.8 ± 18.4	0.108
	Worry (W)	52.4 ± 21.5	57.5 ± 28.2	0.566
	Daily Activities (DA)	46.0 ± 24.7	60.0 ± 21.8	0.127
	Family Relationships (FR)	77.8 ± 23.5	82.0 ± 17.4	0.613
	Total Score (TOT)	65.5 ± 17.9	75.8 ± 15.6	0.123
	Parent HRQL Summary Score (HRQL)	67.5 ± 19.2	79.6 ± 14.8	0.083
	Family Functioning Summary Score (FFSS)	65.9 ± 22.2	73.8 ± 15.0	0.311
Pediatric Quality of Life (PEDSQL-C)	Physical Functioning (PF)	46.7 ± 24.7	46.6 ± 30.6	0.988
	Physical Symptoms (PS)^*^	62.8 ± 16.8	-	-
	Emotional Functioning (EF)	71.7 ± 23.9	76.0 ± 13.3	0.597
	Social Functioning (SF)	60.5 ± 27.3	68.0 ± 19.9	0.438
	Cognitive Functioning (CF)^*^	32.5 ± 37.2	-	-
	School Functioning (SchF)	56.9 ± 18.6	72.8 ± 22.1	0.163
	Psychosocial Health Summary Scale (PsyHSS)	62.5 ± 20.8	72.4 ± 16.1	0.188
	Physical Health Summary Scale (PhyHSS)	48.6 ± 25.0	46.6 ± 30.6	0.842
	Total Score (TOT)	56.7 ± 18.5	63.2 ± 19.8	0.368
Parenting Stress Index-Short Form (PSI-SF)	Parental Distress (PD)	55.4 ± 31.5	42.6 ± 35.7	0.303
	Parent–Child Dysfunctional Interaction (PCDI)	**60.2 ± 23.6**	**41.1 ± 22.6**	**0.036**
	Difficult Child (DC)	54.0 ± 32.9	41.6 ± 29.8	0.310
	Defensive Responding (DR)	47.4 ± 30.1	39.1 ± 35.6	0.489
	Parenting Stress Index-Short Form (PSI-SF) total score	57.6 ± 29.4	41.1 ± 31.9	0.152

Statistically significant values are formatted in bold. ^*^The empty PS and CF cells of the PEDSQL-C correspond to dimensions that cannot be evaluated in the age range of the CG study population.

## 4 Discussion

This study explored the effects of a music therapy intervention during the hospitalization of children with CP, together with their parents. In addition to the intensive neurorehabilitation program, a personalized music therapy project with the EM was implemented. We studied its effect on sleep quality, temperamental characteristics, quality of life, and parental distress.

Our findings showed in the EG improvement in the quality of sleep, particularly in initiating and maintaining sleep (DIMS), and sleep–wake transition disorders (SWTD), not found in the CG.

These findings confirmed the results of our previous study (39).

Previous studies (8, 40) showed that children with CP had more difficulty sleeping than the control group. In particular, the experimental group has more difficulties in initiating and maintaining sleep (DIMS), arousal disturbances (DA), and complications in the sleep–wake transition (SWTD). In our study, which integrates EM into the rehabilitation design, we saw an increase in two of the three impaired sleep areas. In addition, sleep disturbances were negatively correlated with the quality of life of children with CP (41) and their caregivers (8). Therefore, it is very important to evaluate this aspect in the care of children with CP. In support of this, the review by de Almeida et al. underlines how the motor impairment and consequent sleep disturbances of children with CP affect the health of the parent due to the night care required. This shows that high levels of stress, amplified by sleep deprivation, cause a negative impact on the quality of life of the parent and child (8). In addition, a relationship between motor impairment and sleep disturbances was found by Bautista et al. It was observed that children with GMFCS level 5 had more severe sleep disturbances due to the reduced ability to roll in bed, which led them to have more sustained waking times, more pain, and not being able to manage any airway obstructions (42).

The results of our study show that the children improved the quality of their sleep without the use of medication, despite the persistence of their problems. All this led to an improvement in the child’s quality of life and a statistically significant difference in the parent’s quality of life.

In addition, the active role and commitment of the parents, during the EM music therapy sessions, allowed them to acquire new strategies for the affective containment of children in facilitating the transition to sleep.

Even more, we can hypothesize that the specific EM element responsible for this improvement also comes from the creation of custom musical compositions. Listening to and interacting with one’s own sound story (personalized composition) produced a condition of relaxation in the child during and after the music therapy session. Our hypothesis is supported by feedback from some parents, who reported that the effect of relaxation lasted until the following morning.

Therefore, we believe that music therapy coupled with multisensory stimulation has had a great impact on the child and the parent. This could explain the results obtained even with a limited number of therapy sessions. EM music therapy is not only related to the auditory system. It extends its sound action through the implementation of images and tactile sensations that correspond congruously to the representation of sound. For example, if different types of water sounds are played (water droplets, tap water, waterfalls, sea, etc.), the patient, while handling a cushion filled with water, simultaneously sees projected images of the corresponding sound. This allows the activation of memory and attention and promotes emotional involvement.

The behavioral and emotional response of the child to others and the environment, investigated by the QUIT, in EG highlights a statistically significant improvement in the expression of positive emotions, not found in CG. No relevant improvement in EG was observed in attention, as this aspect was not a direct object of music therapy intervention. Furthermore, comparing the results in this subscale at T0, a significant difference emerges between the groups (the CG showed a better score than the EG), while no differences emerge at T1 (at this point we observe a better score in the EG and worse in the CG compared to previous step). This data in EG can be explained by the parent’s learned ability to provide adequate stimulation and capture positive expressions, even minimal, from the child. In addition, we interpreted the statistically significant change in the attention variable of the CG as a possible effect of rehabilitation treatment. Although EG parents reported an improvement in their children’s attention span, which can also be seen in video recordings of EM sessions, paradoxically this field did not emerge from the tests. In our opinion, questionnaires may not be suitable for verifying this.

Results do not show significant improvement in parental distress in EG, as in CG. However, comparing the two groups emerged differences between EG and CG in T0 and T1 in Parent–Child Dysfunctional Interaction (PCDI). Analyzing qualitatively, scores in this sub-scale highlight a slight improvement in the EG compared to a worsening in the CG. These results can be explicated by the capacity of EG parents to care for and manage the critical aspects of their child using new coping strategies acquired during the music therapy training.

Previous studies demonstrated poorer quality of life in mothers and caregivers of children with CP (8, 43, 44), special in social functioning. A study by Glinac et al. (43) demonstrated that the quality of life of CP children’s mothers does not depend on the increase in the child’s motor skills. Comparing this finding with the results that emerged in ours, we could therefore hypothesize that the quality of life of mothers is positively affected by interventions not directly aimed at the physical rehabilitation of the child. Our study showed a significant improvement in the quality of life of the EG family; particularly in the Emotional Functioning, Social Functioning, Worry, and Daily Activities subscales and in the Total Score and Parent HRQL Summary Score. No changes emerge in CG. These results can be explained by the parent’s chance to experience acceptance and discover another key to understanding and managing their child using EM training.

Instead, the PEDSQL-C results documented an improving trend without reaching statistical significance. The trend of EG compared to CG showed improvements in the variables of the Emotional Functioning, Physical Functioning, and the Physical Health Summary Scale. This could be explained by considering the clinical complexity and variability shown by children with CP. The reduced number of case studies could be the reason for not achieving a statistically significant difference.

Many EG parents have reported that their children during EM training were motivated to give motor, communicative, and visual attention performance that did not emerge in other life or rehabilitation contexts. They also reported a positive change in their relationship with their child, which is particularly critical for a child with a disability. However, the assessment scales selected at the start of the study were not sensitive to these parent-reported changes, some of which were unexpected. For this reason, we are currently evaluating new assessment methods that are better suited to detect the changes that emerged during this study.

Despite the improvements observed, some of the investigated variables do not improve, like sleep breathing disorders (SBD) and quality of life of children, because they are directly linked to the organic component compromised by CP. Likewise, in social orientation, motor activity and attention there are no significant improvement, maybe because they are not the main goal of EM music therapy. The EM acts on the caregiver-child interaction; this is probably the reason why the emotional structure of family relationships does not change while other aspects related to the caregiver-child relationship improve in statistically significant way.

Among the biases of the study should be considered the small number of cases studied and the limited time of treatment with EM, related to the duration of hospitalization of the children.

## 5 Conclusion

Our study suggests that music therapy with the Euterpe Method has beneficial effects on fundamental aspects of the child’s and his parents’ lives, such as sleep, emotion control, and quality of family life. These improvements are particularly relevant considering that they occur during a stressful period of hospitalization. Music therapy with the Euterpe Method makes a valuable contribution to the rehabilitation process, creating a context in which the child and parent are an active part of the treatment and the target of the care.

## Data availability statement

The original contributions presented in the study are included in the article/supplementary material, further inquiries can be directed to the corresponding author.

## Ethics statement

Ethical approval was not required for the study involving human samples in accordance with the local legislation and institutional requirements. Written informed consent for participation in this study was provided by the participants’ legal guardians/next of kin. Written informed consent was obtained from the minor(s)’ legal guardian/next of kin for the publication of any potentially identifiable images or data included in this article.

## Author contributions

TL: Conceptualization, Methodology, Writing – original draft, Investigation, Writing – review & editing. SB: Resources, Writing – review & editing. MR: Writing – review & editing, Supervision, Funding acquisition. FD'A: Investigation, Visualization, Writing – original draft, Writing – review & editing. SS: Resources, Writing – review & editing. EN: Investigation, Writing – original draft. MD: Investigation, Writing – original draft. SP: Formal analysis, Writing – original draft, Writing – review & editing, Visualization. RG: Writing – review & editing. EC: Conceptualization, Funding acquisition, Project administration, Supervision, Writing – original draft, Writing – review & editing.

## Glossary

EM: Euterpe method
CP: Cerebral palsy
EG: Experimental group
CG: Control group
GMFCS: Gross Motor Function Classification System
SDSC: Sleep Disturbance Scale for Children
DIMS: Disorders of initiating and maintaining sleep
SBD: Sleep breathing disorders
DA: Disorders of arousal/nightmares
SWTD: Sleep–wake transition disorders
DOES: Disorders of excessive somnolence
SHY: Sleep hyperhidrosis
PEDSQL-C: Pediatric Quality of Life of infants, toddlers, and children
PEDSQL-F: Pediatric Quality of Life of parents
PF: Physical Functioning
PS: Physical Symptoms
EF: Emotional Functioning
SF: Social Functioning
CF: Cognitive Functioning
TOT: Total Score
PsyHSS: Psychosocial Health Summary Score
PhyHSS: Physical Health Summary Score
Com: Communication
W: Worry
DA: Daily Activities
FR: Family Relationships
HRQL: Parent HRQL Summary Score
FFSS: Family Functioning Summary Score
PSI-SF: Parenting Stress Index-Short Form
PSI-SF: Parenting Stress Index-Short Form
PD: Parental distress
PDCI: Parent–child dysfunctional interaction
DC: Difficult child
DR: Defensive responding
